# Identification of serum β-catenin as a biomarker in patients with HBV-related liver diseases

**DOI:** 10.1186/s12967-018-1645-x

**Published:** 2018-09-29

**Authors:** Liang Duan, Qianfan Yang, Jun Yang, Qin Hu, Bo Wang, Pu Li, Weixian Chen

**Affiliations:** 1 Department of Laboratory Medicine, The Second Affiliated Hospital of Chongqing Medical University, No.74 Linjiang Road, Yu Zhong District, Chongqing, 400010 China; 2 Key Laboratory of Diagnostic Medicine Designated by the Chinese Ministry of Education, Chongqing Medical University, Chongqing, 400016 China

**Keywords:** Hepatitis B virus, β-Catenin, Chronic hepatitis, Liver disease

## Abstract

**Background:**

Substantial evidence indicates that β-catenin is a pivotal regulator that contributes to the initiation and development of various types of diseases. Recently, β-catenin can be detected in human serum and also reported to be correlated with several disease progression in a little research. However, very little is known about the relationship between serum β-catenin and HBV-related liver disease.

**Methods:**

Serum levels of β-catenin, from 77 patients with chronic hepatitis B (CHB), 63 patients with hepatitis B associated liver cirrhosis (HBLC), 61 patients with hepatocellular carcinoma (HCC), 41 healthy HBV carriers (HHCs) and 78 healthy controls (HCs) were measured by ELISA. Correlations of serum β-catenin with viral replication and liver necroinflammation parameters were analyzed. The receiver operating characteristic (ROC) curve was used to assess the discriminating power of serum β-catenin to grade different stages of HBV-related disorders. Human hepatic cell line L02 was transfected with a HBV plasmid, and β-catenin levels and the underlying mechanism were analyzed.

**Results:**

Chronic hepatitis B and HBLC patients but not HHC or HCC showed significantly higher serum β-catenin levels than HCs. β-catenin levels were not correlated with HBV DNA levels but were correlated with necroinflammation parameters. HBV-infected cell model showed elevated levels of phosphorylation at Ser473 in Akt (p-Akt), phosphorylation at Ser9 in GSK3β (p-GSK3β) and β-catenin, all of which was blocked by treatment with Akt inhibitor LY294002. Additionally, ROC analysis of β-catenin for discriminating patients with CHB from HHCs, which yielded an AUC of 0.71 (cutoff value, 42 pg/mL; 95% CI 0.61–0.81) with 64.93% sensitivity, 73.17% specificity and 69.05% accuracy. ROC analysis of β-catenin for discriminating patients with HCC from chronic HBV infection mainly including CHB and HBLC, which yielded an AUC of 0.75 (cutoff value, 42 pg/mL; 95% CI 0.67–0.83) with 66.43% sensitivity, 75.41% specificity and 70.92% accuracy.

**Conclusions:**

HBV infection enhances β-catenin expression by activating Akt/GSK3β signaling. Serum β-catenin levels are correlated with necroinflammation parameters but not with viral load. Serum β-catenin has potential to discriminate the phase of HBV-related disorders, particularly predicts the patients with CHB from HHCs and also predicting HCC form chronic HBV infection.

## Background

Hepatitis B virus (HBV) infection is one of the most important infectious diseases in the digestive system, which leads to a wide spectrum of liver disease ranging from acute and chronic hepatitis (CHB), hepatitis B associated liver cirrhosis (HBLC) and hepatocellular carcinoma (HCC). Despite the advent of effective vaccines, as well as other disease control measures, including antiviral therapy, HBV infection is still a serious global health problem [1]. The mechanisms responsible for the liver damage caused by HBV infection are complicated, and both viral and host factors can influence the outcome of HBV infection [2]. Although it has been well established that adaptive immunity plays a critical role in viral clearance, the pathogenetic mechanisms that cause liver damage during HBV infection remain largely unknown.

Wnt/β-catenin signaling pathway is a cell signal transduction pathway that has been linked to a number of disease conditions, including neurodegenerative diseases, psychiatric diseases, cancers, asthma, and even wound healing [3]. The central mediator of the pathway is β-catenin, a multifunctional protein that either can associate with cadherins at the cell membrane to regulate cellular adhesion or can translocate to the nucleus, where it functions as a transcriptional coactivator and modulates hundreds of genes. In the liver disease, activation of Wnt/β-catenin pathway has been identified as a main factor in HCC progression, which is known to be accompanied by accumulation of β-catenin expression within hepatocytes, with its cytoplasmic or nuclear translocation [4]. Previous studies have documented that the HBV X (HBx) protein and hepatitis B surface antigen (HBsAg) can act as pathogenic factors that are involved in the modulation and induction of Wnt/β-catenin pathway and thereby contribute to HBV-induced liver carcinogenesis [5–7]. Several studies have been attempted to use of small molecules targeting the Wnt/β-catenin pathway for potential application for HCC treatment [8, 9]. These publications suggest that Wnt/β-catenin pathway activation may play an important role in the progression of HBV-related liver diseases.

Recently, apart from accumulation of β-catenin expression in cytoplasm or nuclear translocation, β-catenin levels can be detected in human serum and also have been reported to correlate with several disease progression, including hepatitis C-associated hepatocellular carcinoma, type 2 diabetes mellitus, postmenopausal osteoporosis [10–13]. Since previous publications mainly focused on intracellular role of β-catenin during HBV-related disorders, the serum β-catenin and its correlation with disease progression in HBV-related disorders still need to elucidate. However, to date, no research papers have been published which focus on the serum β-catenin levels in HBV-related disorders. Here, serum β-catenin levels in HBV-related liver diseases were measured and their association with disease progression was also analyzed in detail.

## Methods

### Patients and sample collection

Serum samples (n = 320) with HBV-related disorders, healthy HBV carrier and healthy controls were prospectively recruited from the Second Affiliated Hospital of Chongqing Medical University from May 2016 to May 2017. The patients with HBV-related disorders had not received antiviral treatment within 6 months prior to this study’s enrollment. These serum samples were divided into four groups. Group 1 (CHB; n = 77) included patients with chronic hepatitis. Group 2 (HBLC; n = 63) included patients with histological proven hepatitis B associated liver cirrhosis. Group 3 (HCC; n = 61) included patients with histological proven HCC. Group 4 (HHCs; n = 41) included healthy HBV carriers with low replicative (HBV DNA < 2000 IU/mL) and normal ALT. Group 5 (Healthy control; n = 78) included normal healthy subjects with no history of liver disease that were negative for HCV and HBV. The clinicopathological data of the subjects in this study at initial diagnosis were collected, which included gender, age, liver function tests and HBV DNA levels are presented in Table 1. Serum collected from 1 mL of coagulated blood by centrifugation were immediately separated and frozen at − 80 °C until assayed. For tissue samples, clinical histological proven CHB (n = 4), HBLC (n = 6) and HCC (n = 8) samples were collected from patients who underwent liver biopsy at the Second Affiliated Hospital of Chongqing Medical University. Simultaneously, five age and sex matched distal normal tissues from HCC patients who had undergone HCC resection were recruited in this study, and these healthy controls (HCs) were subjected to blood test to rule out the HBV infection. Informed written consent was obtained from all patients and the study was approved by the Institutional Ethics Committee for human studies at the Second hospital affiliated to Chongqing Medical University, Chongqing, China. The clinical characteristics of study subjects are demonstrated in Table 1.

**Table 1:** Clinical characteristics of subjects in this study

Parameter	HC	HHC	CHB	HBLC	HCC
Number of subjects	78	41	77	63	61
Gender (male, %)	45 (57.6%)	29 (70.7%)	50 (64.9%)	47 (74.6%)	39 (63.9%)
Age (range, mean)	19–67, 42.1	17–59, 39	26–66, 45.3	32–68, 48.2	37–67, 53.5
ALT (U/L)	15.8 ± 7.43	16.3 ± 8.33	143.8 ± 144.1	46.11 ± 41.37	16.46 ± 6.56
AST (U/L)	17.98 ± 6.32	17.34 ± 7.91	173.1 ± 254.5	61.52 ± 79.3	21.5 ± 7.25
HBV DNA (log_10_ IU/mL)	N/A	2.58 ± 0.69	5 ± 1.65	4.96 ± 1.7	4.97 ± 2.04

For age, ALT, AST, HBV DNA titres, data are presented as mean ± SD

*N/A* not available

### Plasmid and antibody

pcDNA3.1-HBV plasmid contain 1.3 or 1.1 fold HBV genome fragment were constructed in our laboratory. The antibodies included anti-β-catenin (Santa Cruz Biotechnology, Santa Cruz, CA), anti-glycogen synthase kinase (GSK)-3β, anti-phospho-GSK3β, anti-Akt, anti-phospho-Akt (Cell Signaling Technology, Beverly, MA), anti-β-actin (Boster Biological Technology, California, USA) and Horseradish peroxidase-conjugated anti-mouse, anti-rabbit IgG antibodies (Boster Biological Technology, California, USA).

### Cells culture and transfections

Human hepatic cell line L02 was grown in DMEM (Hyclone, USA) supplemented with 10% heat-inactivated FBS (Lonsera, Uruguay), 100 U/mL penicillin, and 100 U/mL streptomycin. Cell culture was maintained at 37 °C in a humid atmosphere containing 5% CO_2_. Transfection of L02 cells with pcDNA3.1-HBV (1.3 or 1.1) or its control pcDNA3.1 was conducted using lipofectamine 2000 (Invitrogen) according to the manufacturer’s recommendation. And the cells were collected after transfection for indicated time for the subsequent experiment.

### ELISA assay

Serum levels of β-catenin were measured by a commercially available enzyme-linked immunosorbent assay by ELISA kit (CUSABIO, Wuhan, China) according to the manufacturer’s instructions.

### Western blot

Western blot analysis was applied to detected levels of β-catenin, GSK3β, p-GSK3β (Ser9), Akt, p-Akt (Ser473) and β-actin in cells. Briefly, the cells were collected and washed with ice-cold PBS, then lysed on ice in radio immunoprecipitation assay (RIPA) buffer. Samples containing equal amount of proteins were separated in 10% SDS-PAGE and blotted onto the PVDF membranes. Then the membranes were blocked with 5% bovine serum albumin and incubated with anti-β-catenin, anti-GSK3β, anti-p-GSK3β, anti-Akt, anti-p-Akt and β-actin (1:1000 dilution, respectively), followed by incubation with secondary antibodies conjugated with horseradish peroxidase. The proteins of interest were detected using the SuperSignal West Pico Chemiluminescent Substrate kit. The results were recorded by the Bio-Rad Electrophoresis Documentation (Gel Doc 1000, Bio-Rad, USA) and Quantity One Version 4.5.0.

### Immunofluorescence

The cells were plated and cultured onto cleaned-up cover slips, and were washed with phosphate-buffered saline (PBS) and fixed in 4% paraformaldehyde, then permeabilized with 0.2% Triton X-100. Cover slips were rinsed and incubated with blocking serum for 15 min at 37 °C and then incubated with anti-β-catenin antibody (1:100 dilution) overnight at 4 °C. After three washes with PBS, the cells were stained with the corresponding Alexa fluor 647-conjugated antibody. To visualize nuclei, cells were stained with 10 µg/mL DAPI. The fluorescent images were then observed and analyzed using a multilaser confocal microscope.

### Immunohistochemistry (IHC)

For IHC analysis, the sections from the formalin fixed, paraffin-embedded tissues were deparaffinized and rehydrated. Then the sections were boiled for 10 min in 0.01 M citrate buffer and incubated with 0.3% H_2_O_2_ in methanol to block endogenous peroxidase. And the sections were incubated with the anti-β-catenin antibody (1:200 dilution), followed by incubation with secondary antibody tagged with the peroxidase enzyme and were visualized with 0.05% DAB until the desired brown reaction product was obtained. All slides were observed under a Nikon E400 Light Microscope and representative photographs were taken.

### Flow cytometry

Annexin V-PI staining was also used to evaluate the apoptosis of L02 cells. Annexin V-FITC apoptosis kit were purchased from Becton–Dickinson (San Diego, CA). The cells were harvested after treatment, washed twice with pre-chilled PBS and resuspended in 1× binding buffer at a concentration of 1 × 10^6^ cells/mL. One hundred microliter of the cell suspension (1 × 10^5^ cells) was mixed with 5 mL of Annexin V-FITC and 5 μL of propidium iodide according to the manufacturer’s instruction. The mixed solution was gently vortexed and incubated in dark at room temperature (25 °C) for 15 min. Four hundred microliter of 1× dilution buffer was then added to each tube and apoptosis analysis was performed by BD FACS Canto™ II flow cytometer.

### Lactate dehydrogenase (LDH) assay

First of all, cells were transfected with or without plasmids as described above for indicated time in a 6-well plate. After that, the cells were seeded onto a 96-well plate at a density of 1 × 10^4^ cells per well. The release of LDH from cells to the culture medium was detected by a LDH Cytotoxicity Assay Kit (Beyotime, Beijing, China) to evaluate the cytotoxicity. The LDH release was quantified by measuring the UV absorbance at 490 nm.

### Real time quantitative PCR analysis

L02 cells were transfected with pcDNA3.1-HBV (1.3 or 1.1) or pcDNA3.1 for 24 h and then lysed with Trizol (Invitrogen, Carlsbad, CA, USA). Complementary single-stranded DNA was synthesized from total RNA by reverse transcription (TaKaRa, Japan). Primers were also synthesized by Invitrogen. PCR primers were as follows: β-catenin primers: (forward) 5′-CTGCAGGGGTCCTCTGTG-3′ and (reverse) 5′-TGCATATGTCGCCACACC-3′; β-actin primers: (forward) 5′-TCCCTGGAGAAGAGCTACGA-3′ and (reverse) 5′-AGCACTGTGTTGGCGTACAG-3′. Reactions were performed in triplicate using SYBR Green master mix (TaKaRa, Japan) and normalized to GAPDH mRNA level using the ΔΔCt method.

### Statistical analyses

The differences in the results of cells were analyzed using one way ANOVA followed by the Student–Newman–Keuls test, and the differences in the results of serum β-catenin levels were performed using Mann–Whitney test. ROC curves were generated to classify patients in different groups, as well as for the evaluation of the diagnostic potential of serum β-catenin via calculation of the area under the ROC curve (AUC), sensitivity and specificity according to standard formulas. All statistical analyses were performed using GraphPad Prism software (GraphPad Software, CA, USA). Statistical differences are presented at probability levels of p < 0.05, p < 0.01 and p < 0.001.

## Results

### Expression of β-catenin in patients with HBV-relative liver diseases

**Fig. 1:**
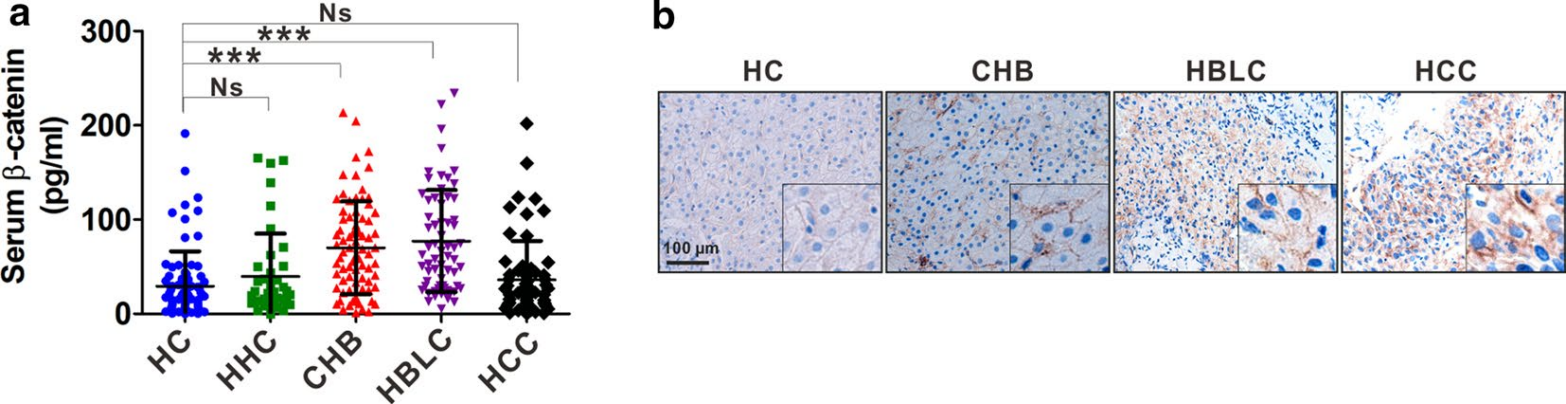
β-catenin expression in serum and tissue samples of HBV-related liver diseases. **a** ELISA analysis of serum β-catenin levels from healthy controls (HCs), healthy HBV carriers (HHCs) and patients with HBV-related liver diseases with different phases (CHB, HBLC and HCC). **b** IHC staining of β-catenin in representative biopsied liver samples from patients with CHB, HBLC and HCC. Blank scale bars = 100 µm. Data represents the mean ± SD. ***p < 0.001. *Ns* no statistical significance

To assess whether serum β-catenin levels are abnormally altered in HBV-relative liver diseases, including CHB, HBLC and HCC, we detected and analyzed its levels in these groups of patients and HCs. CHB and HBLC patients showed significantly higher serum β-catenin levels than HCs (Fig. 1a). While no obvious change was observed in HBV-relative HCC patients (Fig. 1a). Additionally, serum levels of β-catenin in HHCs were also detected and analyzed, which also showed no obvious change compared with HCs (Fig. 1a). We also examined the β-catenin expression in tissue sections from HBV-relative disorders by IHC staining. HC showed a relative low expression of β-catenin expression, which was predominantly distributed on cell membrane of hepatic cells (Fig. 1b). In CHB and HBLC, β-catenin expression was mainly distributed on the membrane as well as existence in the extracellular space (Fig. 1b). And high β-catenin expression was mainly distributed in the cytoplasm of HCC cells (Fig. 1b).

### Relationship between serum β-catenin and viral replication in HBV-related liver diseases

**Fig. 2:**
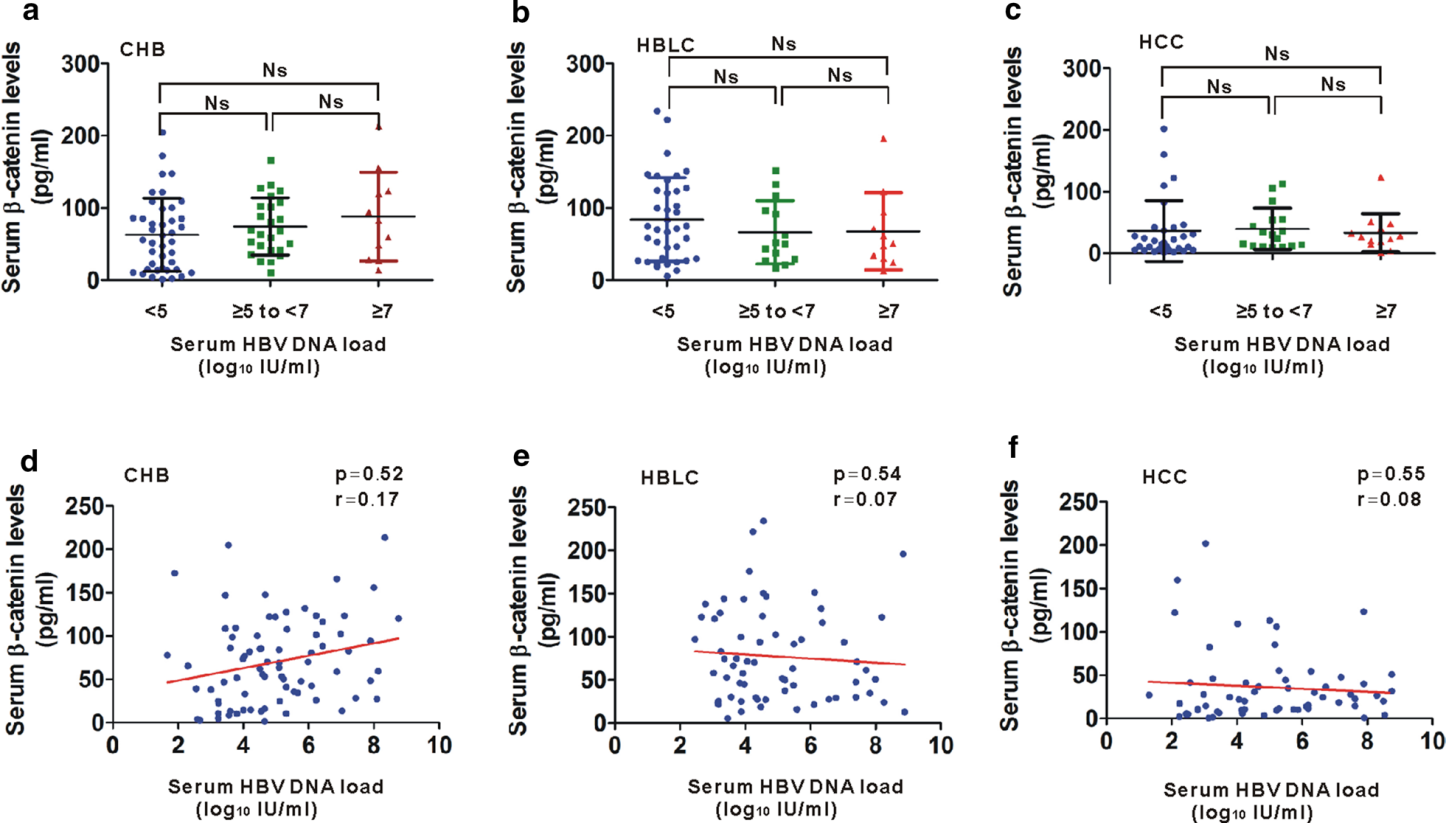
Correlations of serum β-catenin levels with HBV DNA in HBV-related liver diseases. **a**–**c** Distribution of serum β-catenin levels in CHB (**a**), HBLC (**b**) and HCC (**c**) patients with different viral load (< 5, ≥ 5–7 and ≥ 7 log_10_ IU/mL). **d**–**f** Correlation between serum β-catenin levels and HBV DNA levels in CHB (**d**), HBLC (**e**) and HCC (**f**) patients. Data represents the mean ± SD, *Ns* no statistical significance

Based on abnormal serum β-catenin levels in HBV-relative disorders, we then investigate whether serum β-catenin levels are associated with HBV replication. According to HBV DNA levels, CHB patients were classified to three subgroups, including high virus load (≥ 7 log_10_ IU/mL), intermediate virus load (≥ 5–7 log_10_ IU/mL) and low virus load (< 5 log_10_ IU/mL). We found that average levels of serum β-catenin was not proportional to the increase of serum HBV DNA in CHB patients (Fig. 2a). Similar results were also observed in HBLC and HCC patients (Fig. 2b, c). We further analyzed the correlation of serum β-catenin levels with viral loads. β-catenin levels were not found to be correlated with HBV DNA levels in CHB patients (Fig. 2d). Similar phenomenon regarding the relationship between serum β-catenin and HBV DNA levels was also verified in patients with HBLC and HCC patients (Fig. 2e, f).

### Relationship between serum β-catenin and necroinflammation parameters in HBV-related liver diseases

**Fig. 3:**
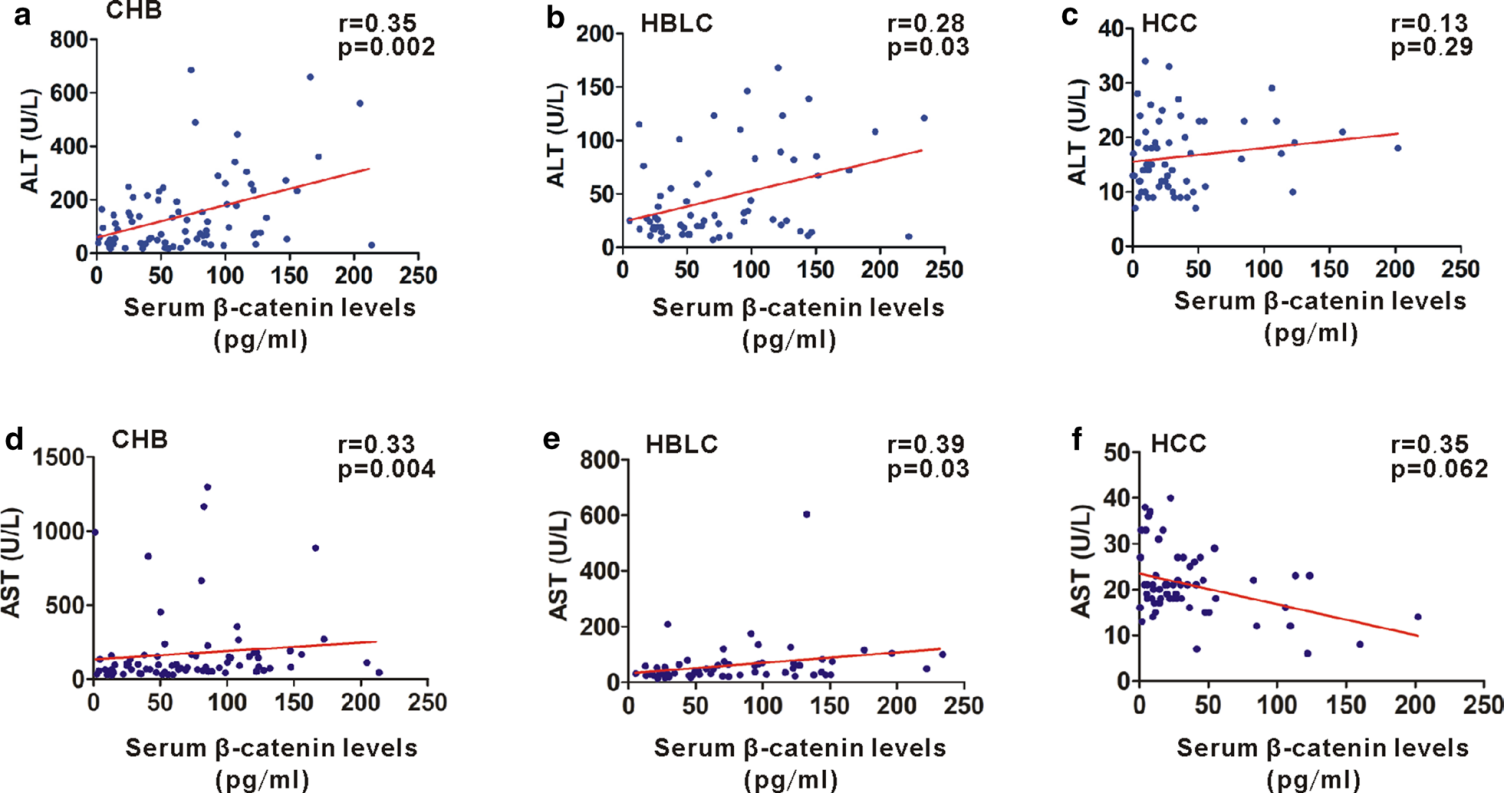
Relationship between serum β-catenin levels and liver necroinflammation. **a**–**c** Correlation between serum β-catenin levels and ALT levels in CHB (**a**), HBLC (**b**) and HCC (**c**) patients. **d**–**f** Correlation between serum β-catenin levels and AST levels in CHB (**d**), HBLC (**e**) and HCC (**f**) patients. *p < 0.05; **p < 0.01; ***p < 0.001

We further analyzed the relationship of serum β-catenin levels with necroinflammation parameters such as ALT and AST in HBV-relative disorders. In this work, we showed that there was a strong significant correlation between serum β-catenin levels and ALT in patients with CHB or HBLC (Fig. 3a, b). However, serum β-catenin levels were not correlated with ALT in HCC patients (Fig. 3c). Additionally, a same phenomenon regarding the relationship between serum β-catenin and AST was also found in HBV-related disorders, showing that a strong significant correlation between serum β-catenin levels and AST in patients with CHB or HBLC but not in HCC (Fig. 3d–f).

### Increased β-catenin expression in HBV-infected hepatic cells

**Fig. 4:**
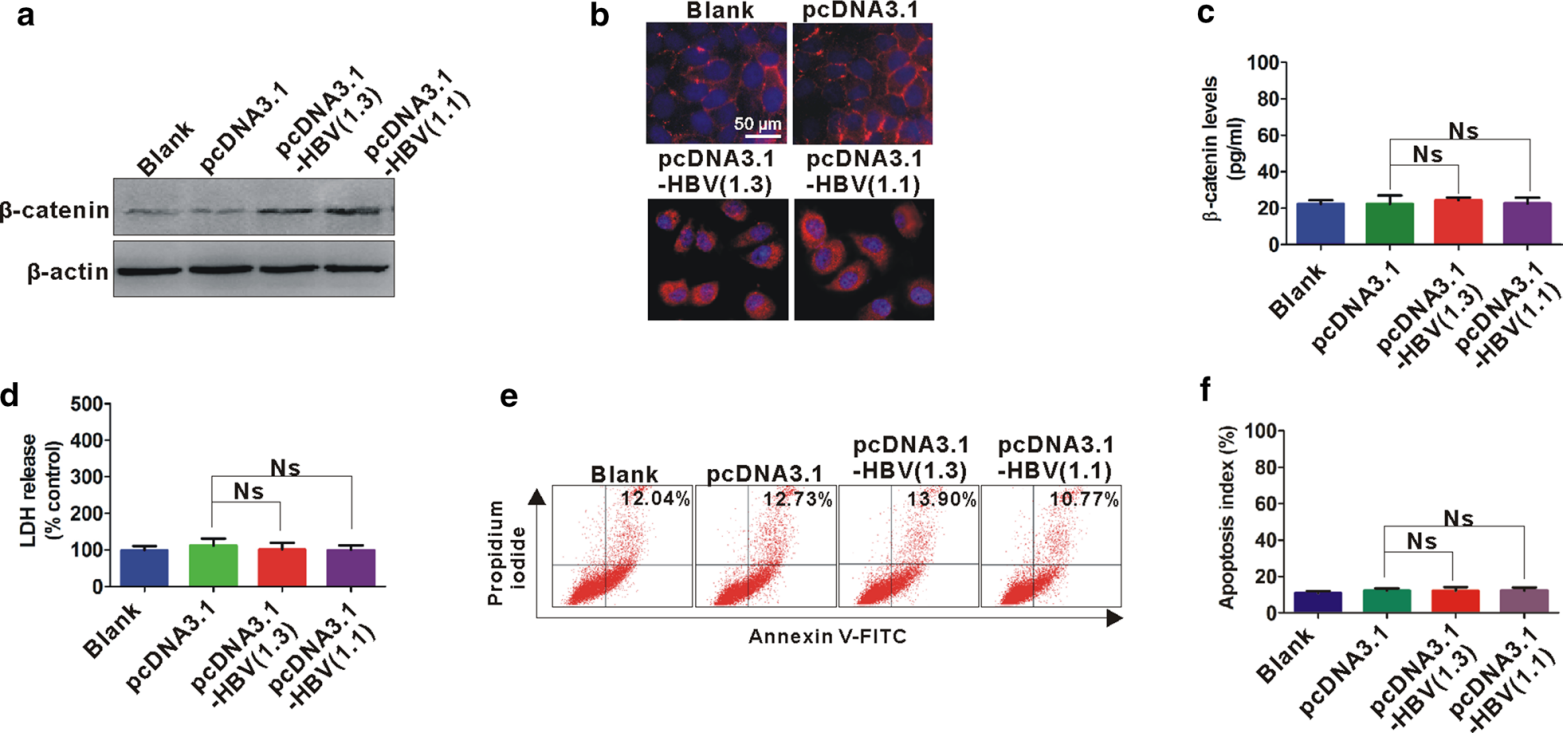
Increased β-catenin expression in HBV-infected hepatic cells. **a** Western blot analysis of β-catenin expression in hepatic L02 cells transfected with and without pcDNA3.1-HBV (1.3 and 1.1) or its control pcDNA3.1 for 48 h. β-actin served as a loading control. **b** Immunofluorescence staining for β-catenin in L02 cells transfected with and without pcDNA3.1-HBV (1.3 and 1.1) or its control pcDNA3.1 for 48 h. White scale bars = 50 µm. **c** ELISA assay for β-catenin expression in L02 cells transfected with and without pcDNA3.1-HBV (1.3 and 1.1) or its control pcDNA3.1 for 48 h. **d** LDH assay for LDH from L02 cells transfected with and without pcDNA3.1-HBV (1.3 and 1.1) or its control pcDNA3.1 for 48 h. **e** Flow cytometry analysis for apoptotic L02 cells transfected with and without pcDNA3.1-HBV (1.3 and 1.1) or its control pcDNA3.1 for 72 h. **f** Apoptosis index for L02 cells with different treatment. *Ns* no statistical significance

HBV 1.3-fold genome plasmid (pcDNA-HBV1.3) and HBV 1.1-fold genome plasmid (pcDNA-HBV1.1) were transfected into human hepatic cell line L02 to establish an HBV-infected hepatic cell model. The protein expression and distribution of β-catenin was analyzed by western blot and immunofluorescence assay, respectively. HBV-infected L02 cells showed an elevated β-catenin expression, which mainly accumulated in the cytoplasm and nucleus (Fig. 4a, b). Additionally, β-catenin levels in cell cultural supernatants were also analyzed by ELISA, which showed no obvious change in cell cultural supernatants of HBV-infected hepatic cell model (Fig. 4c). We also analyzed the cell damage and apoptosis status in HBV-infected hepatic cells by LDH release assay and flow cytometry, respectively. No obvious change of cell damage and apoptosis was found in HBV-infected hepatic cell model (Fig. 4d–f).

### Akt/GSK3β signaling is responsible for elevated β-catenin expression in HBV-infected hepatic cells

**Fig. 5:**
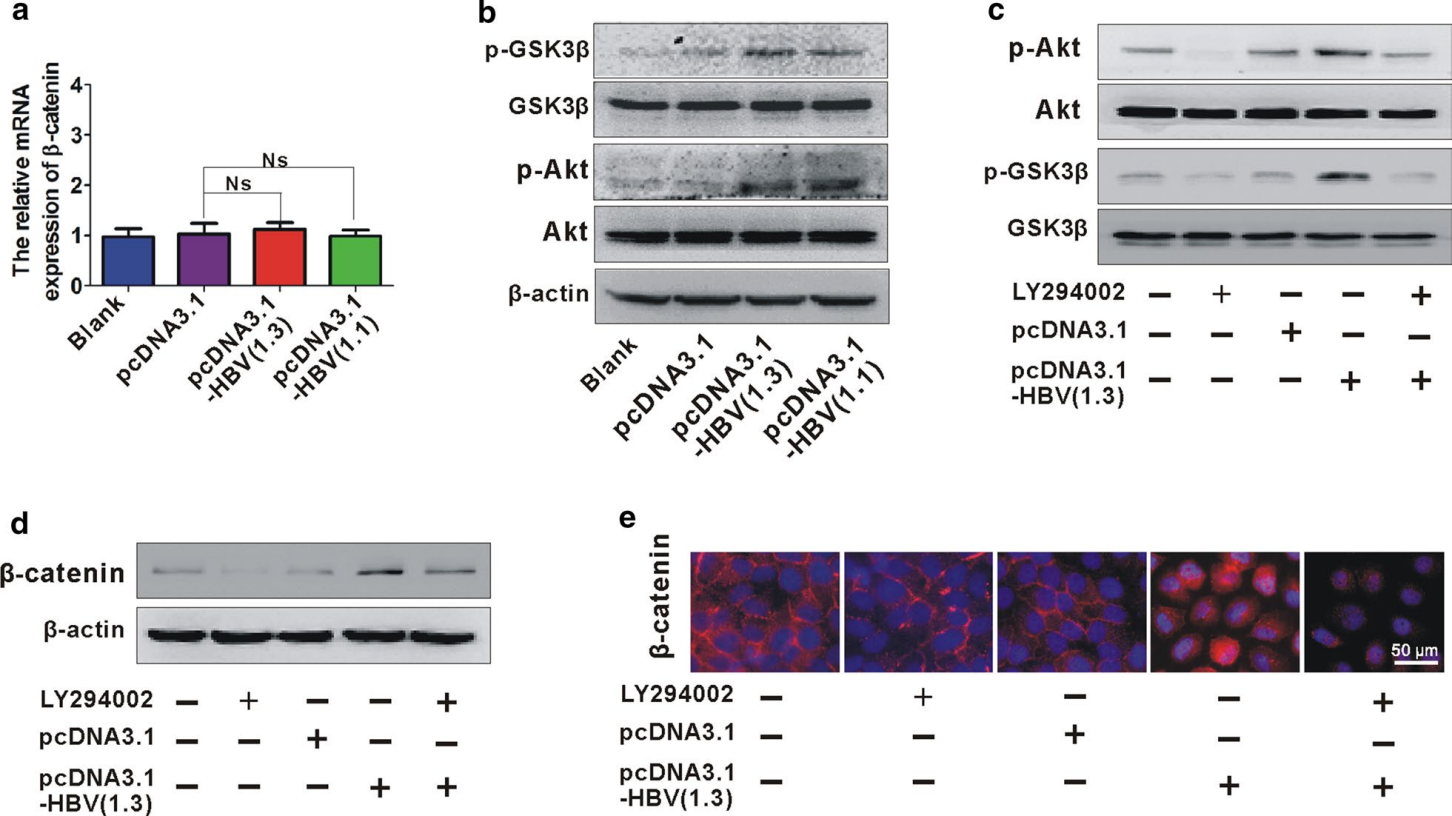
Akt/GSK3β signaling is responsible for elevated β-catenin expression in HBV-infected hepatic cells. **a** Real-time PCR analysis of β-catenin gene in L02 cells transfected with and without pcDNA3.1-HBV (1.3 and 1.1) or its control pcDNA3.1 for 24 h. **b** Western blot analysis of p-GSK3β and p-Akt expression in L02 cells transfected with and without pcDNA3.1-HBV (1.3 and 1.1) or its control pcDNA3.1 for 48 h. **c**, **d** Western blot analysis of p-GSK3β, p-Akt (**c**) and β-catenin (**d**) expression in L02 cells transfected with and without pcDNA3.1-HBV (1.3) or its control pcDNA3.1 followed by treatment with LY294002 (20 μM) for 48 h. **e** Immunofluorescence staining for β-catenin expression in L02 cells transfected with and without pcDNA3.1-HBV (1.3) or its control pcDNA3.1 followed by treatment with LY294002 (20 μM) for 48 h. White scale bars = 50 µm

Enhanced β-catenin was found in HBV-infected hepatic cells. We then explore whether elevated protein levels of β-catenin was resulted by its gene levels. No obvious change of gene levels of β-catenin was observed in HBV-infected hepatic cell model (Fig. 5a), suggesting that elevated β-catenin is not due to its gene levels. β-catenin levels are normally kept low by a phosphorylation process mediated by GSK3β, which targets β-catenin for ubiquitylation and proteasomal degradation [14]. GSK3β phosphorylation at Ser9 resulted in its inactivation and then induces accumulation of β-catenin [15]. So we next investigate whether phosphorylation of GSK3β is involved in HBV-induced accumulation of β-catenin. We detected and analyzed the phosphorylation of GSK3β in cell lysates of HBV-transfected L02 cells by western blot. An increased phosphorylation levels of GSK3β (Ser9) were observed in HBV-infected L02 cells (Fig. 5b). Activated Akt phosphorylates GSK3β, which leads to inactivation of GSK3β and accumulation of β-catenin [16]. We then detected the phosphorylation of Akt (Ser473) and also observed an increased Akt (Ser473) phosphorylation in HBV-infected L02 cells (Fig. 5b). To further confirm activated Akt/GSK3β signaling is responsible for β-catenin accumulation, we use Akt specific inhibitor LY294002 (20 μM) to treat HBV-infected L02 cells and phosphorylation levels of Akt and GSK3β was analyzed. Treatment with LY294002 suppressed increased levels of phosphorylation Akt and GSK3β resulted by HBV (Fig. 5c). Additionally, treatment with LY294002 also suppressed HBV-induced increased levels of β-catenin (Fig. 5d, e). These results suggest that Akt/GSK3β signaling is responsible for HBV-induced β-catenin.

### Differentiating power of β-catenin for progression in HBV-related liver diseases

**Fig. 6:**
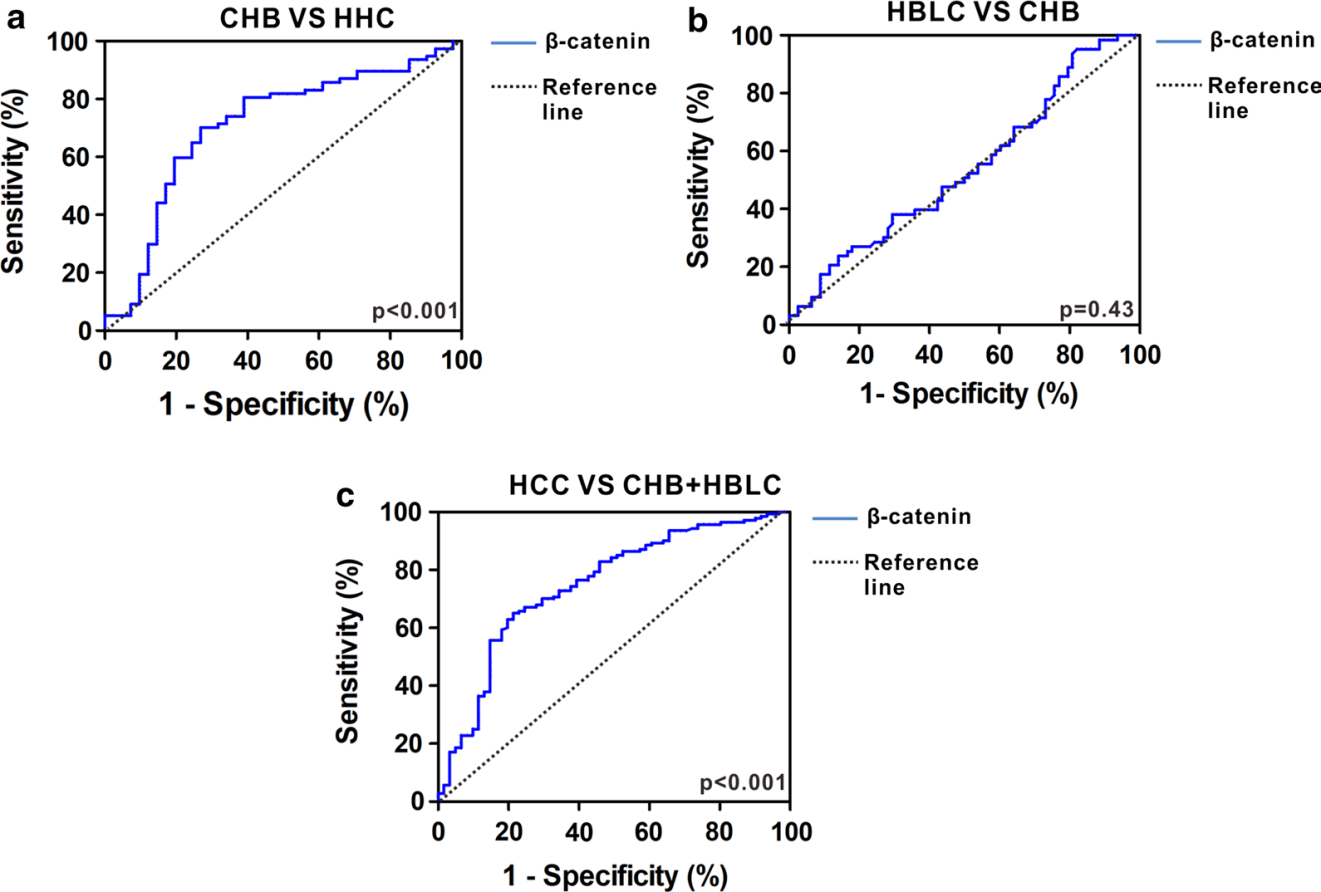
Differentiating power of serum β-catenin for HBV-related liver diseases with different phases. **a** ROC curves of serum β-catenin for detecting CHB patients from HHCs. **b** ROC curve of serum β-catenin for detecting HBLC from CHB patients. **c** ROC curves of serum β-catenin for detecting HCC from chronic HBV infection mainly including CHB and HBLC

We next evaluated differentiating power of β-catenin for progression of HBV-related liver diseases. The ROC analysis indicated that diagnostic value of serum β-catenin yielded an AUC of 0.71 (cutoff value, 42 pg/mL; 95% CI 0.61–0.81) with 64.93% sensitivity, 73.17% specificity and 69.05% accuracy (Fig. 6a). These findings indicate that the identified β-catenin could efficiently discriminate CHB patients from HHCs. To further evaluate whether serum β-catenin can predict HBLC from CHB, we compared serum β-catenin levels between CHB patients and HBLC. ROC analysis showed that it is not able to discriminate between HBLC and CHB patients, which yielded an AUC of 0.53 (95% CI 0.44–0.63) with 47.62% sensitivity, 52.56% specificity and 50.09% accuracy (Fig. 6b). We also determined predictive role of serum β-catenin for HBV-relative HCC from chronic HBV infection mainly including CHB and HBLC. ROC analysis showed that serum β-catenin had better diagnostic value for identifying HBV-relative HCC, which yielded an AUC of 0.75 (cutoff value, 42 pg/mL; 95% CI 0.67–0.83) with 66.43% sensitivity, 75.41% specificity and 70.92% accuracy.

## Discussion

Over the past few decades, an increasing experimental evidence has demonstrated that Wnt/β-catenin pathway is linked to a number of disease conditions, including neurodegenerative diseases, psychiatric diseases, cancers, asthma, and even wound healing [3, 17]. Wnt/β-catenin pathway activation has also been recently identified as a main factor in HBV-induced liver carcinogenesis [4, 18]. The central mediator of the pathway is β-catenin, a multifunctional protein that can translocate to the nucleus, where it functions as a transcriptional coactivator and modulates hundreds of pathogenic genes. Of note, β-catenin can be detected in human serum and also reported to be correlated with several disease progression in a little research and experimentation. Since previous studies mainly focused on intracellular role of β-catenin during HBV-related carcinogenesis, the serum β-catenin levels and its correlation with disease progression in HBV-related disorders still need to elucidate. In our present study, we investigated the association of serum β-catenin with viral replication and liver necroinflammation parameter, as well as the regulatory effect of HBV on β-catenin expression in vitro.

Serum β-catenin levels were assessed in HBV-relative disorders. Elevated serum β-catenin levels were found in CHB and HBLC but not in HHC and HCC. Histological results showed that β-catenin was highly distributed on the membrane of hepatic cells and in the cytoplasm of HCC cells, while β-catenin was found in the extracellular space in CHB and HBLC patient but not in HCC patient, suggesting that β-catenin may be released from hepatic cells into the extracellular space during CHB and HBLC phases. So elevated serum β-catenin levels are consistent with its extracellular distribution in CHB and HBLC patients.

Previous studies showed that with severity of the disease from CHB to HBLC and HCC, the HBV load gradually decreases [19, 20]. To explore whether elevated β-catenin in CHB and HBLC is caused by high HBV loads during the phases, we analyzed their relationships. Our present data reveal that average levels of serum β-catenin was not proportional to the increase of serum HBV DNA, and no correlation between serum β-catenin and HBV DNA levels was observed in HBV-relative disorders.

Previous study reported that liver injury severity decreased progressively from CHB to HBLC and HCC [21]. Serum β-catenin levels were also decreased progressively from CHB and HBLC to HCC and were positively correlated with ALT or AST in CHB and HBLC phases. We speculate that elevated serum β-catenin is linked to hepatic cell necrosis during CHB and HBLC phases. Interestingly, high intracellular β-catenin levels were found in a transient HBV infection cell model but its levels in the extracellular supernatant were not changed, which is not consistent with its distribution in clinical samples. Generally, HBV is noncytopathic for infected hepatocytes, but the main culprit for liver damage during HBV infection is primarily the adaptive immune attack to virus-infected hepatic cells [22, 23]. In our cellular data, no obvious cell death was found in a transient in vitro HBV infection cell model by LDH release assay and FACS because the cell model is just an in vitro experiment and lack of HBV-induced immune attack. Therefore, intact cell membrane in in vitro HBV infection cell model may not be conducive to the releasing of β-catenin into the extracellular space. In our further studies, releasing of β-catenin into the extracellular space mediated by hepatic cell necrosis during HBV infection would be investigated by using an in vivo HBV infection model.

In our present study, β-catenin was mainly expressed in nucleus and cytoplasm of L02 cells, but it was not expressed in nucleus of patient specimens. Previous study has been reported that β-catenin can translocated from the cytoplasm and nucleus to the plasma membrane in response to high cell density [24]. Here, we speculate that the inconsistency of subcellular regions for β-catenin between in patient liver specimens and in L02 cells may be resulted by the cell density. Patient liver specimens showed a high cell density presented by tight junctions of hepatic cells, but L02 cells used in our in vitro study is scattered with a low cell density.

Four proteins have been identified that directly promote the degradation of intracellular β-catenin: GSK3β, Axin, APC, and β-TrCP/Slimb. These proteins comprise the destruction complex and function to maintain low steady-state levels of β-catenin in the cell. Axin and APC act as scaffolds, binding both β-catenin and GSK3β and facilitating the phosphorylation of β-catenin by GSK3β [25]. The central player in the destruction complex is GSK3β which is thought to phosphorylate one or more conserved serine and threonine residues in the amino-terminal region of β-catenin (S33, S37, T41, and S45) [26]. In our present study, GSK3β phosphorylation at Ser9 (inactivation form) were observed in HBV-infected L02 cells, suggesting that HBV infection may inactivate GSK3β. There is no evidence that GSK3β can be combined with HBV or its subgroup members. We then focus on the upstream classical regulatory factor Akt, which has been able to be activated by HBV or its subgroup member HBX [27, 28]. Activated Akt was also found in HBV-infected L02 cells and suppression of Akt or GSK3β also blocked the β-catenin accumulation, suggesting that Akt/GSK3β signaling is involved in the HBV-induced β-catenin accumulation.

A unique characteristic of β-catenin is that it can be detected in human serum where they are correlated with a few disease progression. Here, differentiating power of β-catenin for progression of HBV-related liver diseases was analyzed and we found that serum β-catenin could efficiently discriminate CHB patients from HHC, while it is not able to discriminate between HBLC and CHB patients. ROC analysis also showed that serum β-catenin levels had predictive role for HBV-relative HCC from chronic HBV infection mainly including CHB and HBLC. All these evidences suggest that β-catenin might be the future candidate marker for diagnosis of HBV-related liver diseases. Recently, in addition to HBV infection, activation of β-catenin signaling has also been verified in other viral infections, including herpesvirus, influenza A and HIV [29–31]. Therefore, pending additional work on larger samples of various types of viral infectious diseases, usage of serum levels of β-catenin for diagnosis is promising.

Our study has several limitations. First, we did not evaluate the specificity of serum β-catenin for HBV infection. It would be better to compare HBV infection with other disease entities such as chronic hepatitis C or nonalcoholic steatohepatitis using the same set of analysis. Second, our study was carried out in Chinese patients who were characterized by Genotype B and C, further studies are required in CHB patients from different geographical areas or with different genotypes to confirm these data.

## Conclusions

In this study, we provide strong evidence that patients with CHB and HBLC but not with HHC and HBV-related HCC harbor increased levels of serum β-catenin. Importantly, we have shown that (i) serum β-catenin levels correlate with liver necroinflammation and may predict the patients with CHB from HHC and also predict HBV-related HCC form chronic HBV infection (CHB and HBLC) in clinical samples. By using the HBV-infected cell model, we confirm that Akt/GSK3β signaling activation mediates accumulation of β-catenin. Therefore, our data support the notion that serum β-catenin may be a useful tool for assessing HBV-related liver diseases.

## Abbreviations

CHB: chronic hepatitis B
HBLC: hepatitis B associated liver cirrhosis
HCC: hepatocellular carcinoma
HCs: healthy controls
GSK: glycogen synthase kinase
ROC: receiver operating characteristic
AUC: area under the curve
HBsAg: hepatitis B surface antigen
RIPA: radio immunoprecipitation assay
IHC: immunohistochemistry

## References

[1] Yuen MF, Chen DS, Dusheiko GM, Janssen HLA, Lau DTY, Locarnini SA, Peters MG, Lai CL (2018). Hepatitis B virus infection. Nat Rev Dis Prim.

[2] Oh IS, Park SH (2015). Immune-mediated liver injury in hepatitis B virus infection. Immune Netw.

[3] Nusse R, Clevers H (2017). Wnt/beta-catenin signaling, disease, and emerging therapeutic modalities. Cell.

[4] Levrero M, Zucman-Rossi J (2016). Mechanisms of HBV-induced hepatocellular carcinoma. J Hepatol.

[5] Zhang XD, Wang Y, Ye LH (2014). Hepatitis B virus X protein accelerates the development of hepatoma. Cancer Biol Med.

[6] Tian X, Li J, Ma ZM, Zhao C, Wan DF, Wen YM (2009). Role of hepatitis B surface antigen in the development of hepatocellular carcinoma: regulation of lymphoid enhancer-binding factor 1. J Exp Clin Cancer Res.

[7] Daud M, Rana MA, Husnain T, Ijaz B (2017). Modulation of Wnt signaling pathway by hepatitis B virus. Arch Virol.

[8] Vilchez V, Turcios L, Marti F, Gedaly R (2016). Targeting Wnt/beta-catenin pathway in hepatocellular carcinoma treatment. World J Gastroenterol.

[9] Chen J, Rajasekaran M, Hui KM (2017). Atypical regulators of Wnt/beta-catenin signaling as potential therapeutic targets in hepatocellular carcinoma. Exp Biol Med (Maywood).

[10] Thiele A, Wasner M, Muller C, Engeland K, Hauschildt S (2001). Regulation and possible function of beta-catenin in human monocytes. J Immunol.

[11] Tian J, Xu XJ, Shen L, Yang YP, Zhu R, Shuai B, Zhu XW, Li CG, Ma C, Lv L (2015). Association of serum Dkk-1 levels with beta-catenin in patients with postmenopausal osteoporosis. J Huazhong Univ Sci Technol Med Sci.

[12] Gaudio A, Privitera F, Battaglia K, Torrisi V, Sidoti MH, Pulvirenti I, Canzonieri E, Tringali G, Fiore CE (2012). Sclerostin levels associated with inhibition of the Wnt/beta-catenin signaling and reduced bone turnover in type 2 diabetes mellitus. J Clin Endocrinol Metab.

[13] Zekri AR, Bahnassy AA, Alam El-Din HM, Morsy HM, Shaarawy S, Moharram NZ, Daoud SS (2011). Serum levels of beta-catenin as a potential marker for genotype 4/hepatitis C-associated hepatocellular carcinoma. Oncol Rep.

[14] Metcalfe C, Bienz M (2011). Inhibition of GSK3β by Wnt signalling—two contrasting models. J Cell Sci.

[15] Ding Q, Xia W, Liu JC, Yang JY, Lee DF, Xia J, Bartholomeusz G, Li Y, Pan Y, Li Z (2005). Erk associates with and primes GSK-3beta for its inactivation resulting in upregulation of beta-catenin. Mol Cell.

[16] Lee JE, Kang JS, Ki YW, Lee SH, Lee SJ, Lee KS, Koh HC (2011). Akt/GSK3beta signaling is involved in fipronil-induced apoptotic cell death of human neuroblastoma SH-SY5Y cells. Toxicol Lett.

[17] Steinhart Z, Angers S (2018). Wnt signaling in development and tissue homeostasis. Development.

[18] Cavard C, Colnot S, Audard V, Benhamouche S, Finzi L, Torre C, Grimber G, Godard C, Terris B, Perret C (2008). Wnt/beta-catenin pathway in hepatocellular carcinoma pathogenesis and liver physiology. Future Oncol.

[19] Kataki K, Borthakur P, Kumari N, Deka M, Kataki AC, Medhi S (2017). Association of mRNA expression of toll-like receptor 2 and 3 with hepatitis B viral load in chronic hepatitis, cirrhosis, and hepatocellular carcinoma. J Med Virol.

[20] Pei YZ, Han T, Ma XY, Li Y, Xing J, Song ZL (2011). The discrepancy of HBsAg titre and HBV DNA in patients with chronic hepatitis B, HBV-related liver cirrhosis and hepatocellular carcinoma. Zhonghua Gan Zang Bing Za Zhi.

[21] Hoan NX, Khuyen N, Binh MT, Giang DP, Van Tong H, Hoan PQ, Trung NT, Anh DT, Toan NL, Meyer CG (2016). Association of vitamin D deficiency with hepatitis B virus—related liver diseases. BMC Infect Dis.

[22] Dienes HP, Drebber U (2010). Pathology of immune-mediated liver injury. Dig Dis.

[23] Guidotti LG, Chisari FV (2006). Immunobiology and pathogenesis of viral hepatitis. Annu Rev Pathol.

[24] Dietrich C, Scherwat J, Faust D, Oesch F (2002). Subcellular localization of beta-catenin is regulated by cell density. Biochem Biophys Res Commun.

[25] Miller JR, Hocking AM, Brown JD, Moon RT (1999). Mechanism and function of signal transduction by the Wnt/beta-catenin and Wnt/Ca^2+^ pathways. Oncogene.

[26] Wu D, Pan W (2010). GSK3: a multifaceted kinase in Wnt signaling. Trends Biochem Sci.

[27] Rawat S, Bouchard MJ (2015). The hepatitis B virus (HBV) HBx protein activates AKT to simultaneously regulate HBV replication and hepatocyte survival. J Virol.

[28] Zhu M, Li W, Lu Y, Dong X, Lin B, Chen Y, Zhang X, Guo J, Li M (2017). HBx drives alpha fetoprotein expression to promote initiation of liver cancer stem cells through activating PI3K/AKT signal pathway. Int J Cancer.

[29] Kumar A, Zloza A, Moon RT, Watts J, Tenorio AR, Al-Harthi L (2008). Active beta-catenin signaling is an inhibitory pathway for human immunodeficiency virus replication in peripheral blood mononuclear cells. J Virol.

[30] Zhu L, Thunuguntla P, Liu Y, Hancock M, Jones C (2017). The beta-catenin signaling pathway stimulates bovine herpesvirus 1 productive infection. Virology.

[31] Hillesheim A, Nordhoff C, Boergeling Y, Ludwig S, Wixler V (2014). beta-catenin promotes the type I IFN synthesis and the IFN-dependent signaling response but is suppressed by influenza A virus-induced RIG-I/NF-kappaB signaling. Cell Commun Signal.

